# Clinical study on Knee Three Needles combined with ultra-short wave for knee osteoarthritis

**DOI:** 10.1097/MD.0000000000050448

**Published:** 2026-09-04

**Authors:** Xueqin Yu, Minghua Yuan, Mengxin Yi, Hong Ding, Jun Huang, Wenbing Zou, Xiaoyan Yan

**Affiliations:** a Department of Rehabilitation Medicine, Yichun People’s Hospital, Yichun City, Jiangxi Province, China; b Department of Internal Medicine, The Second Affiliated Hospital of Yichun University, Yichun City, Jiangxi Province, China.

**Keywords:** combination therapy, knee osteoarthritis, Knee Three Needles, Lysholm score, TCM syndrome type, ultra-short wave

## Abstract

This study investigates the clinical efficacy of Knee Three Needles combined with ultra-short wave for knee osteoarthritis and its effects on different traditional Chinese medicine syndrome types. This retrospective, non-randomized cohort study enrolled 180 patients with knee osteoarthritis from June 2024 to June 2025. Patients were classified according to the rehabilitation protocols previously received into 3 groups (n = 60 each): Knee Three Needles (Group A), ultra-short wave (Group B), and combination therapy (Group C). All received basic treatment (education, exercises, and oral glucosamine hydrochloride). Group A received Knee Three Needles acupuncture (main points: bilateral Knee Eye, Liangqiu, and Xuehai; adjunct points based on syndrome). Group B received ultra-short wave therapy (nonthermal/microthermal, 20 minutes/session). Group C received both treatments. All interventions were administered once daily, with 10 sessions per course. Individual matching for age (±3 years) and body mass index (±1.5) was applied, with analysis of covariance correcting for baseline differences. Visual Analog Scale (rest/motion pain), Lysholm score, and knee range of motion (ROM) were assessed pre- and posttreatment. Baseline data were comparable among groups (*P* > .05). All groups showed significant posttreatment improvements in Visual Analog Scale, Lysholm score, and ROM (*P* < .001). Group C demonstrated significantly lower posttreatment rest pain (1.68 ± 0.95) and motion pain (2.95 ± 1.36) versus Groups A and B (*P* < .05), with superior pain reduction (*P* < .01). Lysholm score (80.26 ± 10.42) and improvement value (31.30 ± 10.68) in Group C were significantly higher than those in the other groups (*P* < .01). Flexion/extension improvements in Group C (18.32 ± 9.56°/5.19 ± 3.42°) also surpassed those in Groups A and B (*P* < .01). The total efficacy rate in Group C (93.3%) was significantly higher than that in Group A (83.3%) and that in Group B (78.3%; *P* < .05). Subgroup analysis revealed significantly greater Lysholm score improvements in Group C across Blood Stasis, Dampness, and Cold Predominance syndromes versus the monotherapy groups within the same syndrome types (*P* < .05 or *P* < .01). For Cold Predominance syndrome, the improvement in Group A (23.18 ± 9.24) exceeded that in Group B (18.28 ± 9.56). In this retrospective cohort, receipt of Knee Three Needles combined with ultra-short wave was associated with greater short-term improvements in pain, function, and ROM than either monotherapy. Because treatment allocation was non-randomized, these findings should be interpreted as associations rather than proof of causality or synergy.

## 1. Introduction

Knee osteoarthritis (KOA) is a chronic joint disease characterized by degenerative changes in articular cartilage and secondary osteophyte formation. Its main clinical manifestations include knee pain, swelling, and limited mobility, which in severe cases, can lead to joint deformity and dysfunction, seriously affecting patients’ quality of life. Epidemiological surveys indicate that the prevalence of symptomatic KOA in China is 8.1%, showing an increasing trend with age and a higher prevalence in females than in males.^[1]^ With accelerated population aging, KOA has become a significant public health issue, imposing substantial economic burdens on individuals, families, and society.

Current treatment for KOA follows a stepwise principle. Basic treatment includes patient education, lifestyle guidance, functional exercises, and pharmacotherapy.^[2]^ When basic treatment proves insufficient, physical therapy, acupuncture, intra-articular injections, or surgical interventions may be selected based on the condition severity. Ultra-short wave therapy, a commonly used physical modality in clinical practice, acts on deep joint tissues through high-frequency electric fields, producing thermal effects that promote local blood circulation, reduce inflammatory responses, and alleviate pain and stiffness.^[3]^ Acupuncture for KOA has a long history with confirmed clinical efficacy and is recommended by multiple guidelines.^[4]^ “Jin’s Three Needle Therapy” represents a representative academic thought of the Lingnan acupuncture school, among which “Knee Three Needles” (bilateral Knee Eye, Liangqiu, and Xuehai) serves as a classic point combination for knee joint disorders, functioning to dredge meridians, harmonize Qi and blood, and facilitate joint movement.^[5]^ In clinical practice, Knee Three Needles are often modified with additional points according to traditional Chinese medicine (TCM) syndrome differentiation, reflecting the characteristic advantages of TCM treatment based on pattern identification.

In recent years, integrated traditional Chinese and Western medicine treatment for KOA has gained increasing attention. The combined application of acupuncture and physical therapy may produce synergistic effects through different mechanisms of action. The thermal effect of ultra-short wave can improve local joint microcirculation, creating favorable conditions for acupuncture to dredge meridians, while the regulatory effect of acupuncture can enhance the anti-inflammatory and analgesic effects of ultra-short wave.^[6]^ Although clinical studies on Knee Three Needles or ultra-short wave alone for KOA have been reported, clinical evidence for their combined application remains insufficient, and few studies have explored the efficacy differences of combined therapy across different TCM syndrome types from the perspective of TCM pattern identification.

Based on this, the present study adopted a retrospective cohort design, controlling for confounding factors through individual matching, to compare short-term clinical outcomes among patients who received Knee Three Needles, ultra-short wave, or their combination for KOA, and further explore outcome differences among patients with different TCM syndrome types. This study aims to provide preliminary observational evidence for clinical treatment selection in KOA and a basis for integrated traditional Chinese and Western medicine treatment of KOA.

## 2. Methods

### 2.1. Study subjects and grouping

This study was approved by the Ethics Committee of Yichun People’s Hospital. This study adopted a retrospective cohort design, collecting data from patients with KOA who received rehabilitation treatment in our hospital from June 2024 to June 2025. Based on different rehabilitation treatment protocols, patients were divided into 3 groups: Group A (Knee Three Needles group), Group B (ultra-short wave group), and Group C (combination therapy group).

Treatment allocation was not randomized and was not determined by the investigators for study purposes. Group membership reflected rehabilitation protocols that patients had already received in routine clinical care; therefore, the study evaluates associations between treatment exposure and outcomes rather than causal treatment effects.

### 2.2. Confounding factor matching

To reduce selection bias, this study employed restrictive individual matching combined with statistical correction to control for confounding factors:

1. Individual matching: To ensure basic comparability of core clinical indicators, individual matching was performed for age (±3 years) and body mass index (±1.5). Eligible patients were screened from the 3 groups, ultimately including 60 cases in each group.
2. Statistical correction: After matching, gender, disease course, affected side, Kellgren–Lawrence (K-L) grade, comorbidities (hypertension and diabetes), smoking history, and pretreatment Visual Analog Scale (VAS)/Western Ontario and McMaster Universities Osteoarthritis Index scores were compared among the 3 groups. If statistically significant differences (*P* < .05) or clinically meaningful imbalances (e.g., substantial differences in disease course) existed in these indicators between groups, such variables were included as covariates in multiple regression models or analysis of covariance (ANCOVA) during subsequent efficacy comparisons to eliminate their confounding effects on outcome measures.
3. Residual confounding: Matching and covariate adjustment were used to improve baseline comparability but cannot fully account for unmeasured or imperfectly measured factors in a retrospective observational study. Accordingly, adjusted between-group differences were interpreted as associations.

### 2.3. Inclusion and exclusion criteria

#### 2.3.1. Inclusion criteria

Patients meeting all the following conditions were included: age ≥40 years, regardless of gender; meeting the diagnostic criteria for KOA established in the “Osteoarthritis Diagnosis and Treatment Guidelines” by the Orthopedics Branch of the Chinese Medical Association; unilateral or bilateral knee joint involvement; for bilateral cases, the more severely affected side (based on higher K-L grade or VAS pain score) was selected as the observation target; no medication treatment for KOA (e.g., oral nonsteroidal anti-inflammatory drugs and intra-articular injections) or other rehabilitation treatments (e.g., acupuncture and physical therapy) within 1 month before enrollment, based on medical record documentation; and complete clinical medical records available for extracting comprehensive baseline data and efficacy evaluation indicators.

#### 2.3.2. Exclusion criteria

Exclusion criteria were as follows: patients with malignant tumors, severe cardiovascular or cerebrovascular diseases, or hepatic/renal insufficiency; patients with cardiac pacemakers; patients with unremoved metal foreign bodies (e.g., internal fixation devices and metal prostheses); pregnant or lactating women; patients with coagulation disorders or a definite bleeding tendency; patients with other joint diseases such as rheumatoid arthritis, gouty arthritis, infectious arthritis, or traumatic arthritis; and patients with incomplete medical records missing key information (e.g., treatment protocols and efficacy evaluation indicators).

### 2.4. Treatment interventions

All 3 groups received basic treatment under the guidance of the stepwise principle for KOA, including patient education (disease awareness, self-management), lifestyle guidance (avoiding weight-bearing, reasonable rest), scientifically standardized joint and muscle functional exercises (e.g., quadriceps isometric contractions, straight leg raises), and routine oral glucosamine hydrochloride capsules (1 capsule each time, 3 times daily for 10 consecutive days).

#### 2.4.1. Group A (Knee Three Needles group)

Patients received Knee Three Needles acupuncture treatment. Acupuncture needles: Huacheng brand sterile acupuncture needles (manufactured by Beijing Keyuanda Medical Supplies Factory, Registration Certificate No.: Jingxie Zhun 20172271110, Specification: 0.18 × 40 mm). Acupoint selection referred to the “Jin’s Three Needle Clinical Point Matching Method.” Main points: Jin’s Knee Three Needles (bilateral Knee Eye, Liangqiu, and Xuehai). Additional points based on TCM syndrome differentiation: Blood Stasis syndrome: add Geshu, Yanglingquan; Dampness Predominance syndrome: add Zusanli, Yinlingquan; Cold Predominance syndrome: add Shenshu, Guanyuan; Heat Bi syndrome: add Dazhui, Quchi. Procedure: After routine disinfection of local acupoint areas, Knee Eye points were obliquely inserted medially 1.2 to 1.5 cun until Deqi sensation was achieved; Liangqiu and Xuehai were perpendicularly inserted 1.2 cun until Deqi sensation was achieved; additional points received conventional acupuncture with reinforcing and reducing methods applied according to deficiency or excess conditions. Bilateral Knee Eye, Liangqiu, and Xuehai were connected to an electroacupuncture device, using a continuous wave, with current intensity adjusted to patient tolerance, retaining needles for 20 to 30 minutes. Treatment was administered once daily, with 10 sessions constituting 1 course.

#### 2.4.2. Group B (ultra-short wave group)

Patients received ultra-short wave physiotherapy. Equipment: DL-C-M ultra-short wave therapy machine manufactured by Shantou Medical Equipment Factory, output power of 200 W, operating frequency of 40.68 MHz. Patients were placed in the supine position with the affected knee joint exposed. Two electrodes (20 × 14 cm) were placed in superior-inferior or medial-lateral opposition on both sides of the knee joint, with a skin distance of 2 to 3 cm. Treatment dosage: nonthermal or microthermal (adjusted to the patient’s sensation of comfortable warmth). Treatment was administered once daily for 20 minutes, with 10 sessions constituting 1 course.

#### 2.4.3. Group C (combination therapy group)

Patients received both Knee Three Needles acupuncture treatment (as in Group A) and ultra-short wave physiotherapy (as in Group B). Knee Three Needles treatment and ultra-short wave therapy could be administered with an interval between them. Specific operational parameters and acupoint selection were identical to those in Groups A and B. Each treatment was administered once daily, with 10 sessions constituting 1 course.

All 3 groups of patients completed all 10 treatments as planned, demonstrating good treatment compliance.

Treatment exposure differed across groups: the scheduled modality time was approximately 20 to 30 minutes per session in Group A (needle-retention period), 20 minutes per session in Group B, and 40 to 50 minutes per session in Group C because both modalities were delivered (excluding preparation and any interval between modalities). Thus, the combination group had greater total procedure/therapist exposure, which may itself contribute to nonspecific treatment effects.

Operator identity and years of professional experience were not included as standardized variables in the retrospective dataset used for this analysis; therefore, the study cannot reliably establish that all sessions were delivered by the same acupuncturist/therapist or quantify operator-experience effects. This potential provider-related variability is considered in the limitations.

### 2.5. Data collection

Patient data were collected through the hospital’s electronic medical record system. All data were independently verified and entered by 2 researchers to ensure accuracy and completeness, specifically including the following:

1. Demographic characteristics: age, gender, body mass index.
2. Disease characteristics: disease course (months), affected side (left knee/right knee/both knees), K-L imaging grade (grade II, grade III, and grade IV). TCM syndrome differentiation types were classified according to the “Jin’s Three Needle Clinical Point Matching Method” based on patient clinical symptoms into 4 types: Blood Stasis syndrome, Dampness Predominance syndrome, Cold Predominance syndrome, and Heat Bi syndrome.
3. Comorbidities and lifestyle: history of hypertension, history of diabetes, smoking history, alcohol consumption history.
4. Efficacy indicators were assessed before and after treatment:
  a. Pain intensity: Assessed using the VAS. A 10 cm sliding scale was used, with ends representing “no pain” (0 points) and “severe pain” (10 points). Patients marked the position representing pain intensity on the scale based on their subjective feeling, and the score was recorded. This study assessed pain at rest (rest pain) and pain during weight-bearing walking (motion pain) separately.
  b. Knee joint function: Assessed using the Lysholm Knee Score scale. This scale has a total score of 100 points, including 8 dimensions: limp (5 points), support (5 points), locking (15 points), instability (25 points), pain (25 points), swelling (10 points), stair climbing (10 points), and squatting (5 points). Higher scores indicate better knee joint function.
  c. Joint range of motion (ROM): A universal goniometer was used to measure the active ROM of the affected knee joint. Patients were placed in the prone position, with the goniometer axis aligned with the fibular head of the knee, the stationary arm along the femoral axis pointing to the greater trochanter, and the moving arm along the tibial axis pointing to the lateral malleolus. Active flexion and active extension were measured separately. Extension was expressed as negative values indicating the degree of extension limitation (e.g., −5° indicates 5° extension deficit).
  d. Efficacy evaluation criteria: Evaluated based on the improvement rate of the Lysholm score. Improvement rate = (posttreatment score − pretreatment score)/pretreatment score × 100%. Specific grading criteria were as follows:

Controlled: Joint function returned to normal, pain completely disappeared after treatment, Lysholm score improvement ≥85%;
Markedly effective: Joint movement no longer restricted, pain basically disappeared, Lysholm score improvement 60% to 84%;
Effective: Mild joint restriction, pain basically disappeared, Lysholm score improvement 30% to 59%;
Ineffective: Failed to meet the above indicators.

$$\begin{array}{c} \begin{array}{cc} Total\;ef\;fective\;rate=\left(Controlled+Markedly\;ef\;fective+Ef\;fective\right)\\ & \;cases/Total\;cases\;\times 100\%. \end{array} \end{array}$$

The Lysholm Knee Score was treated as the principal functional outcome because it was the functional scale consistently available for retrospective extraction across the included records and it captures multiple domains relevant to knee symptoms and activity (pain, swelling, limp, support, instability, stair climbing, and squatting). However, it is not an osteoarthritis-specific instrument, and this limitation is acknowledged in the Discussion.

1. Safety reporting: The retrospective data collection was primarily designed around efficacy outcomes and did not use a standardized prospective adverse-event case-report form. Consequently, a reliable incidence or between-group comparison of procedure-related adverse events could not be calculated, and safety was not analyzed as a formal endpoint.

### 2.6. Statistical analysis

SPSS Statistics, version 26.0 (IBM Corp.) statistical software was used for data analysis. Normality of data distribution was tested first. Measurement data conforming to a normal distribution were expressed as mean ± standard deviation ($\bar{x}\pm s$), while those not conforming to a normal distribution were expressed as median (interquartile range). Count data were expressed as number of cases (%). For baseline comparisons, one-way ANOVA was used for measurement data conforming to a normal distribution and homogeneity of variance among the 3 groups; otherwise, the Kruskal–Wallis *H* test was employed. The chi-square test was used for count data. For intragroup comparisons before and after treatment, the paired *t* test was used for measurement data conforming to a normal distribution, while the Wilcoxon signed-rank test was used for those not conforming to a normal distribution. For comparisons of efficacy indicators among the 3 groups after treatment, ANCOVA was used with pretreatment scores as covariates to adjust for baseline differences, and pairwise comparisons were performed using the Bonferroni method. Comparisons of efficacy among the 3 groups within different TCM syndrome types also used ANCOVA. Comparisons of total effective rates and efficacy grade distributions among the 3 groups were performed using the chi-square test. The significance level was α = 0.05, and *P* < .05 was considered statistically significant.

Because this was a non-randomized observational analysis, statistical adjustment was intended to reduce measured confounding and was not considered capable of eliminating residual confounding or establishing causality.

## 3. Results

### 3.1. Comparison of baseline data

A total of 180 patients with KOA were included in this study, with 60 cases in each group. After individual matching, there were no statistically significant differences among the 3 groups in demographic characteristics, disease characteristics, distribution of TCM syndrome types, comorbidities, or pretreatment condition (*P* > .05), indicating balanced and comparable baseline data. The mean age ranged from 58 to 60 years, with females accounting for approximately two-thirds. The median disease course ranged from 28 to 40 months, with K-L grades predominantly II and III. The most common TCM syndrome types were Dampness Predominance syndrome and Blood Stasis syndrome. Pretreatment VAS scores ranged from 5.95 to 6.61 points, and Lysholm scores ranged from 48.96 to 52.36 points (see Table 1 for details).

**Table 1:** Comparison of baseline data after matching among the 3 groups ($\bar{x}\pm \;s$).

Parameter	Group A (Knee Three Needles, n = 60)	Group B (ultra-short wave, n = 60)	Group C (combination therapy, n = 60)	Statistic	*P* value
Demographic characteristics					
Age (yr)	58.35 ± 8.62	59.92 ± 8.17	58.68 ± 8.93	*F* = 0.514	.599
Gender (M/F, n)	22/38	26/34	20/40	χ^2^ = 1.283	.527
BMI (kg/m^2^)	24.86 ± 2.93	25.51 ± 2.68	24.93 ± 3.12	*F* = 0.896	.410
Disease characteristics					
Disease course (mo)	28.00 (15.00, 48.00)	36.00 (18.00, 54.00)	40.00 (20.00, 60.00)	*H* = 4.856	.088
Affected side (L/R/B, n)	24/30/6	26/28/6	22/32/6	χ^2^ = 0.647	.957
Kellgren–Lawrence grade (n, %)				χ^2^ = 0.712	.949
Grade II	27 (45.0)	25 (41.7)	26 (43.3)		
Grade III	25 (41.7)	26 (43.3)	26 (43.3)		
Grade IV	8 (13.3)	9 (15.0)	8 (13.3)		
TCM syndrome type (n, %)				χ^2^ = 1.526	.958
Blood Stasis	18 (30.0)	16 (26.7)	17 (28.3)		
Dampness Predominance	20 (33.3)	22 (36.7)	19 (31.7)		
Cold Predominance	14 (23.3)	15 (25.0)	16 (26.7)		
Heat Bi	8 (13.3)	7 (11.7)	8 (13.3)		
Main comorbidities (n, %)					
Hypertension	16 (26.7)	19 (31.7)	21 (35.0)	χ^2^ = 0.961	.619
Diabetes	9 (15.0)	11 (18.3)	13 (21.7)	χ^2^ = 0.877	.645
Lifestyle (n, %)					
Smoking history	12 (20.0)	15 (25.0)	17 (28.3)	χ^2^ = 1.146	.564
Alcohol consumption	10 (16.7)	12 (20.0)	14 (23.3)	χ^2^ = 0.812	.666
Pretreatment condition					
VAS score (points)	5.95 ± 1.46	6.48 ± 1.52	6.61 ± 1.59	*F* = 2.873	.059
Lysholm score (points)	52.36 ± 10.45	50.82 ± 11.23	48.96 ± 11.86	*F* = 2.568	.079

BMI = body mass index, TCM = traditional Chinese medicine, VAS = Visual Analog Scale.

### 3.2. Comparison of pain intensity: analysis of VAS score changes before and after treatment in the 3 groups

To evaluate the analgesic effects of different treatment regimens on pain in patients with KOA, VAS scores for rest pain and motion pain were analyzed before and after treatment in the 3 groups. Before treatment, baseline VAS scores for rest pain and motion pain were balanced among the 3 groups (*P* > .05), indicating comparability. After treatment, rest pain and motion pain scores in all 3 groups decreased significantly compared with those before treatment (*P* < .001). Further intergroup comparison showed that rest pain and motion pain scores in the combination therapy group were significantly lower than those in the Knee Three Needles group and the ultra-short wave group after treatment (*P* < .05), and the pain improvement values were also significantly superior to those of the 2 single-treatment groups (*P* < .01), as detailed in Table 2.

**Table 2:** Comparison of VAS pain scores before and after treatment among the 3 groups (points, $\bar{x}\pm \;s$).

Group	n	Before treatment	After treatment	Intragroup comparison		Pain improvement value	Intergroup comparison (posttreatment)	
				*t* value	*P* value		*F* value	*P* value
Rest pain								
Group A (Knee Three Needles)	60	3.85 ± 1.26	2.12 ± 1.08	12.356	<.001	1.73 ± 1.12		
Group B (ultra-short wave)	60	3.92 ± 1.34	2.35 ± 1.21	10.284	<.001	1.57 ± 1.18	3.892*	.022
Group C (combination therapy)	60	3.78 ± 1.31	1.68 ± 0.95†	15.673	<.001	2.10 ± 1.24‡		
Motion pain								
Group A (Knee Three Needles)	60	6.25 ± 1.46	3.68 ± 1.42	13.892	<.001	2.57 ± 1.38		
Group B (ultra-short wave)	60	6.48 ± 1.52	4.12 ± 1.58	11.456	<.001	2.36 ± 1.45	4.215*	.016
Group C (combination therapy)	60	6.61 ± 1.59	2.95 ± 1.36†	16.784	<.001	3.66 ± 1.52‡		

VAS = Visual Analog Scale.

* Analysis of covariance (ANCOVA) with the pretreatment score as a covariate.

† Compared with Groups A and B, *P* < .05.

‡ Compared with Groups A and B, *P* < .01.

### 3.3. Comparison of knee joint function

To evaluate the effects of different treatment regimens on functional recovery of the knee joint in patients with KOA, Lysholm scores were statistically analyzed before and after treatment in the 3 groups. Before treatment, baseline Lysholm scores were balanced among the 3 groups (*P* > .05), indicating comparability. After treatment, Lysholm scores in all 3 groups increased significantly compared with those before treatment (*P* < .001). Further intergroup comparison showed that the posttreatment Lysholm score in the combination therapy group (80.26 ± 10.42) was significantly higher than that in the Knee Three Needles group (72.48 ± 11.26) and the ultra-short wave group (68.94 ± 12.38; *P* < .05), and the functional improvement value (31.30 ± 10.68) was also significantly superior to those of the 2 single-treatment groups (*P* < .01), as detailed in Table 3.

**Table 3:** Comparison of Lysholm knee scores before and after treatment among the 3 groups (points, $\bar{x}\pm \;s$).

Group	n	Before treatment	After treatment	Intragroup comparison		Functional improvement value	Intergroup comparison (posttreatment)	
				*t* value	*P* value		*F* value	*P* value
Lysholm total score (0–100 points)								
Group A (Knee Three Needles)	60	52.36 ± 10.45	72.48 ± 11.26	15.892	<.001	20.12 ± 8.96		
Group B (ultra-short wave)	60	50.82 ± 11.23	68.94 ± 12.38	12.784	<.001	18.12 ± 9.34	6.234*	.002
Group C (combination therapy)	60	48.96 ± 11.86	80.26 ± 10.42†	18.456	<.001	31.30 ± 10.68‡		

* Analysis of covariance (ANCOVA) with the pretreatment score as a covariate.

† Compared with Groups A and B, *P* < .05.

‡ Compared with Groups A and B, *P* < .01.

### 3.4. Comparison of ROM: analysis of knee flexion and extension changes before and after treatment in the 3 groups

Before treatment, baseline knee flexion and extension ranges were balanced among the 3 groups (*P* > .05). After 10 treatment sessions, flexion degrees improved to varying extents in all 3 groups, and extension limitations were significantly alleviated (*P* < .001). Notably, the combination therapy group demonstrated superior advantages in the magnitude of ROM improvement: the flexion improvement value reached (18.32 ± 9.56)°, substantially exceeding those in the Knee Three Needles group (9.86 ± 8.24)° and the ultra-short wave group (8.56 ± 7.92)°; the extension improvement value (5.19 ± 3.42)° was also superior to those in the 2 single-treatment groups (3.42 ± 2.68)° and (3.04 ± 2.84)°. Intergroup comparisons showed that all indicators in the combination therapy group were significantly better than those in the other 2 groups (*P* < .05 or *P* < .01), as detailed in Table 4.

**Table 4:** Comparison of knee range of motion before and after treatment among the 3 groups (°, $\bar{x}\pm \;s$).

Group	n	Before treatment	After treatment	Intragroup comparison		Range of motion improvement value	Intergroup comparison (posttreatment)	
				*t* value	*P* value		*F* value	*P* value
Active flexion								
Group A (Knee Three Needles)	60	98.56 ± 12.34	108.42 ± 11.56	7.892	<.001	9.86 ± 8.24		
Group B (ultra-short wave)	60	97.82 ± 13.08	106.38 ± 12.24	6.784	<.001	8.56 ± 7.92	4.123*	.018
Group C (combination therapy)	60	96.94 ± 13.45	115.26 ± 10.86†	10.234	<.001	18.32 ± 9.56‡		
Active extension								
Group A (Knee Three Needles)	60	−6.84 ± 3.56	−3.42 ± 2.86	8.456	<.001	3.42 ± 2.68		
Group B (ultra-short wave)	60	−7.12 ± 3.84	−4.08 ± 3.12	7.234	<.001	3.04 ± 2.84	3.892*	.022
Group C (combination therapy)	60	−7.35 ± 4.02	−2.16 ± 2.34†	9.893	<.001	5.19 ± 3.42‡		

Negative extension values indicate the degree of extension limitation (e.g., −5° indicates a 5° extension deficit).

* Analysis of covariance (ANCOVA) with the pretreatment range of motion as a covariate.

† Compared with Groups A and B, *P* < .05.

‡ Compared with Groups A and B, *P* < .01.

### 3.5. Comparison of clinical efficacy: overall efficacy evaluation after treatment in the 3 groups

Clinical efficacy was assessed based on the improvement rate of the Lysholm score. Results showed that the combination therapy group had a control rate of 21.7% and a markedly effective rate of 41.7%, both higher than those in the Knee Three Needles group (11.7%, 35.0%) and the ultra-short wave group (8.3%, 31.7%). The ineffective rate in the combination therapy group was only 6.7%, significantly lower than the 16.7% and 21.7% in the 2 single-treatment groups. Regarding the total effective rate, the combination therapy group reached 93.3%, significantly superior to the Knee Three Needles group (83.3%) and the ultra-short wave group (78.3%). The chi-square test revealed that the overall distribution of efficacy among the 3 groups was statistically significant (*P* < .05), and all indicators in the combination therapy group were significantly better than those in the other 2 groups (*P* < .05), as detailed in Table 5.

**Table 5:** Comparison of clinical efficacy among the 3 groups (n [%]).

Group	n	Controlled	Markedly effective	Effective	Ineffective	Total effective rate (%)	χ^2^ value	*P* value
Group A (Knee Three Needles)	60	7 (11.7)	21 (35.0)	22 (36.7)	10 (16.7)	83.3		
Group B (ultra-short wave)	60	5 (8.3)	19 (31.7)	23 (38.3)	13 (21.7)	78.3	8.234	.016*
Group C (combination therapy)	60	13 (21.7)†	25 (41.7)†	18 (30.0)	4 (6.7)†	93.3†		

* Comparison of efficacy distribution among the 3 groups using a chi-square test showed a statistically significant overall difference (*P* < .05).

† Compared with Groups A and B, *P* < .05.

### 3.6. Subgroup analysis: improvement of Lysholm score after treatment in patients with different TCM syndrome types

To investigate the efficacy differences of Knee Three Needles with syndrome-based point selection across different syndrome types, stratified analysis of Lysholm score improvement was performed within each syndrome type among the 3 groups. Baseline scores were balanced among the 3 groups within each syndrome type before treatment (*P* > .05). Results showed that the improvement values in the combination therapy group for Blood Stasis syndrome, Dampness Predominance syndrome, and Cold Predominance syndrome were (31.67 ± 10.24), (29.60 ± 10.86), and (32.91 ± 10.86) points, respectively, all significantly superior to those in the Knee Three Needles group and the ultra-short wave group within the same syndrome type (*P* < .05 or *P* < .01). Notably, for Cold Predominance syndrome, the improvement value in the Knee Three Needles group (23.18 ± 9.24) was also higher than that in the ultra-short wave group (18.28 ± 9.56), reflecting the syndrome differentiation advantage of acupuncture in warming Yang and dissipating Cold. For Heat Bi syndrome, no statistically significant difference in improvement values was found among the 3 groups (*P* > .05), as detailed in Table 6.

**Table 6:** Comparison of Lysholm score improvement after treatment in patients with different syndrome types (points, $\bar{x}\pm \;s$).

Syndrome type	Group	n	Before treatment*	After treatment	Improvement value	Intragroup comparison		Intergroup comparison†	
						*t* value	*P* value	*F* value	*P* value
Blood Stasis	Group A (Knee Three Needles)	18	48.26 ± 9.84	70.28 ± 10.86	22.02 ± 8.96	8.234	<.001		
	Group B (ultra-short wave)	16	47.53 ± 10.26	66.34 ± 11.42	18.81 ± 9.24	7.126	<.001	5.234	.009
	Group C (combination therapy)	17	46.89 ± 10.58	78.56 ± 9.86‡	31.67 ± 10.24§	9.892	<.001		
Dampness Predominance	Group A (Knee Three Needles)	20	53.42 ± 10.26	73.26 ± 11.24	19.84 ± 8.86	7.892	<.001		
	Group B (ultra-short wave)	22	51.86 ± 11.34	69.86 ± 12.36	18.00 ± 9.12	6.784	<.001	4.892	.012
	Group C (combination therapy)	19	50.24 ± 11.86	79.84 ± 10.68‡	29.60 ± 10.86§	8.956	<.001		
Cold Predominance	Group A (Knee Three Needles)	14	51.68 ± 11.42	74.86 ± 10.92	23.18 ± 9.24	6.892	<.001		
	Group B (ultra-short wave)	15	49.96 ± 10.86	68.24 ± 11.86	18.28 ± 9.56	5.784	<.001	6.234	.004
	Group C (combination therapy)	16	48.35 ± 11.24	81.26 ± 10.24‡	32.91 ± 10.86§	8.234	<.001		
Heat Bi	Group A (Knee Three Needles)	8	58.26 ± 8.96	71.86 ± 9.86	13.60 ± 7.86	4.892	<.001		
	Group B (ultra-short wave)	7	56.84 ± 9.42	70.24 ± 10.42	13.40 ± 8.12	4.234	.002	2.234	.124∥
	Group C (combination therapy)	8	55.68 ± 9.86	73.26 ± 9.68	17.58 ± 8.46	5.234	<.001		

* Pretreatment Lysholm scores for each syndrome type fully correspond to baseline data in Table 1.

† Intergroup comparison using analysis of covariance (ANCOVA) with the pretreatment score as a covariate.

‡ Compared with Groups A and B within the same syndrome type, *P* < .05.

§ Compared with Groups A and B within the same syndrome type, *P* < .01.

∥ The small sample size for Heat Bi syndrome limited statistical power for intergroup comparison (*P* > .05).

## 4. Discussion

The results of this study show that Knee Three Needles, ultra-short wave, and their combination can effectively alleviate pain symptoms and improve knee joint function and ROM in patients with KOA. The efficacy of the combination therapy group was superior to that of the monotherapy groups, suggesting that the combined application of Knee Three Needles and ultra-short wave may have a synergistic effect. This finding is generally consistent with previous related studies. Some clinical research has reported that acupuncture combined with physical therapy can enhance the clinical efficacy for KOA,^[7]^ and meta-analyses have also suggested that integrated traditional Chinese and Western medicine may be superior to monotherapy in relieving pain and improving function for KOA.^[5,8]^ This study evaluated the clinical value of combined therapy from multiple dimensions, including rest pain, motion pain, Lysholm score, and joint ROM, providing a reference basis for clinical practice.

The choice and interpretation of the Lysholm score also require qualification. The scale was selected because it was consistently documented in the retrospective records and provides a multidomain summary of knee symptoms and activity, but it was not developed specifically for KOA. A KOA-specific minimal clinically important difference (MCID) for Lysholm after nonsurgical rehabilitation has not been firmly established. Published studies in other knee populations have reported Lysholm MCID estimates of approximately 4.2 to 10.5 points. The mean 31.30-point improvement observed in the combination group exceeds that published range; however, because those thresholds were derived from different patient populations and interventions, this cross-population comparison should be considered supportive rather than definitive evidence of clinical importance. Future prospective KOA studies should prespecify an OA-specific patient-reported outcome such as the Western Ontario and McMaster Universities Osteoarthritis Index or Knee Injury and Osteoarthritis Outcome Score and a validated clinically important change threshold.

Regarding pain relief, the rest pain and motion pain scores in the combination therapy group after treatment were lower than those in the 2 monotherapy groups, with more pronounced pain improvement values. Mechanistic studies on acupuncture analgesia indicate that acupuncture stimulation may activate the endogenous analgesic system, promoting the release of analgesic substances such as enkephalin and β-endorphin, while also potentially regulating local inflammatory factor levels to exert anti-inflammatory and analgesic effects.^[9,10]^ The thermal effect of ultra-short wave may elevate local tissue temperature, improve blood circulation, accelerate the clearance of pain-causing substances, and alleviate muscle spasm.^[11]^ When combined, the neurohumoral regulatory effect of acupuncture and the physical thermal effect of ultra-short wave may synergize, thereby enhancing analgesic efficacy. The improvement in motion pain was greater than that in rest pain in the combination therapy group, suggesting that combined therapy may have advantages in pain associated with functional activities, which is of positive significance for improving patients’ daily living abilities.

Regarding knee joint function improvement, the Lysholm score improvement value in the combination therapy group was higher than those in the Knee Three Needles group and the ultra-short wave group. The Lysholm score encompasses multiple dimensions, including limp, support, locking, instability, pain, swelling, stair climbing, and squatting, providing a comprehensive reflection of knee joint functional status.^[12]^ The advantages of combined therapy may be related to the following factors: effective pain relief may create conditions for functional exercise; acupuncture may regulate muscle tension around the joint, improving biomechanical balance^[13]^; ultra-short wave may promote absorption of intra-articular inflammation, reduce synovial proliferation, and delay cartilage degeneration.^[14]^ The combination of these 2 modalities may achieve simultaneous improvement in pain and function.

In terms of ROM, the flexion improvement value and extension improvement value in the combination therapy group were higher than those in the monotherapy groups. Knee flexion limitation primarily affects movements such as squatting and stair climbing, while extension limitation may lead to limping during walking.^[15]^ The improvement in ROM with combined therapy was consistent with the improvement in patients’ subjective feelings, further supporting its clinical value. From the perspective of TCM theory, among the Knee Three Needles, the Knee Eye point is an extra meridian point traditionally considered effective for treating knee pain; Liangqiu is the Xi-cleft point of the Stomach Meridian of Foot-Yangming, with the function of dredging meridians and activating collaterals; Xuehai belongs to the Spleen Meridian of Foot-Taiyin, traditionally considered to nourish and activate blood. The combination of these 3 points achieves the effect of dredging local meridian Qi and blood.^[16]^ The thermal effect of ultra-short wave shares similarities with the TCM method of “warming and unblocking,” potentially assisting Yang Qi, dispersing Cold-Dampness, and unblocking meridians. The combination of these 2 modalities may reflect the treatment approach of integrating acupuncture and physical therapy, addressing both manifestations and root causes.^[17]^

Subgroup analysis revealed that the advantages of combination therapy were more pronounced in Blood Stasis syndrome, Dampness Predominance syndrome, and Cold Predominance syndrome, while no statistically significant difference was found among the 3 groups for Heat Bi syndrome. This finding may offer some reference value for clinical treatment selection. Blood Stasis syndrome is characterized by fixed stabbing pain aggravated at night; the effect of ultra-short wave in improving microcirculation may contribute to activating blood and resolving stasis,^[18]^ while acupuncture at Geshu and Yanglingquan enhances the effect of promoting Qi and activating blood.^[19]^ Dampness Predominance syndrome is characterized by swelling and heaviness; acupuncture at Zusanli and Yinlingquan is traditionally considered to strengthen the spleen and resolve dampness,^[20]^ and ultra-short wave may promote absorption of local edema,^[18]^ potentially producing synergistic effects. Cold Predominance syndrome is characterized by aggravation with cold and relief with warmth; acupuncture at Shenshu and Guanyuan is traditionally considered to warm Yang and disperse cold, and the thermal effect of ultra-short wave may directly warm and unblock the local area,^[21]^ thus, the effect of combined therapy may be more prominent. In Cold Predominance syndrome, the improvement value in the Knee Three Needles group was also higher than that in the ultra-short wave group, possibly reflecting the syndrome differentiation advantage of acupuncture in warming Yang and dispersing cold. Heat Bi syndrome is characterized by joint redness, swelling, heat, and pain. In this study, ultra-short wave was administered at nonthermal or microthermal doses with weak thermal effects, and the heat-clearing effect of acupuncture at Dazhui and Quchi may be limited^[22]^; additionally, the sample size was small. Therefore, no intergroup differences were demonstrated, suggesting that for patients with Heat Bi syndrome, combined therapy may need to be applied cautiously in clinical practice, or adjustments to the ultra-short wave dosage and acupuncture point selection protocol may be considered.

Clinical efficacy evaluation showed that the total effective rate and the rate of controlled plus markedly effective cases in the combination therapy group were higher than those in the monotherapy groups. Combined therapy may not only improve the overall effective rate but also increase the proportion of excellent and good outcomes, suggesting that more patients may achieve the therapeutic goals of basic recovery of joint function and basic disappearance of pain.^[21]^ From a health economics perspective, although the single-session cost of combined therapy is slightly higher, the better efficacy may reduce the number of subsequent treatments and medication use, offering potential cost-effectiveness advantages. However, this requires further economic evaluation for confirmation.

This study has several limitations. First, the retrospective, non-randomized design is inherently vulnerable to selection bias, indication bias, information bias, and residual confounding. Although individual matching and ANCOVA improved balance for measured variables, they cannot account for all unmeasured or imperfectly measured factors, such as treatment preference, clinician selection of rehabilitation protocol, daily activity level, previous treatment exposure, adherence outside the clinic, expectations, and psychological factors. Therefore, the observed between-group differences represent associations and should not be interpreted as causal effects or definitive evidence of synergy. Second, the combination group received greater total procedure and therapist-contact time (approximately 40–50 minutes of scheduled modality time per session vs 20–30 minutes in the acupuncture group and 20 minutes in the ultra-short wave group). The study design cannot separate the specific effect of combining modalities from nonspecific effects related to treatment intensity, attention, or time. Third, operator identity and years of professional experience were not standardized analytical variables in this retrospective dataset, so provider-related variability could not be quantified or adjusted. Fourth, safety was not captured with a standardized prospective adverse-event framework, precluding reliable incidence estimates or between-group safety comparisons. Fifth, Lysholm was the functional outcome consistently available in the records but is not KOA-specific, and a validated MCID for nonsurgical KOA rehabilitation is not firmly established; cross-population MCID comparisons should therefore be interpreted cautiously. Sixth, although the matched sample included 60 patients per group, subgroup sizes – especially for Heat Bi syndrome – were small, limiting statistical power and increasing uncertainty around syndrome-specific findings. Seventh, only short-term outcomes after 10 treatment sessions were available, with no long-term follow-up to assess durability, delayed adverse events, or subsequent treatment use. Eighth, retrospective documentation of TCM syndrome differentiation may be incomplete or variable. Finally, this was a single-center study, which limits generalizability to other populations, practitioners, and healthcare settings. Prospective multicenter randomized controlled trials with standardized treatment providers, matched attention/time controls, prespecified KOA-specific patient-reported outcomes, systematic adverse-event monitoring, and longer follow-up are needed.

In summary, in this retrospective cohort, patients who received Knee Three Needles combined with ultra-short wave therapy had greater short-term improvements in pain, knee function, and ROM than patients who received either modality alone. The observed associations appeared more pronounced in Blood Stasis, Dampness Predominance, and Cold Predominance syndromes. Because treatment allocation was non-randomized and the combination group had greater treatment exposure, the results should not be interpreted as definitive evidence of causality or therapeutic synergy. These findings are hypothesis-generating and support prospective, multicenter, adequately powered randomized controlled trials with standardized provider characteristics, attention/time-matched comparators, systematic safety monitoring, KOA-specific functional outcomes, and extended follow-up.

## Author contributions

**Conceptualization:** Xueqin Yu, Minghua Yuan, Mengxin Yi, Hong Ding, Jun Huang, Wenbing Zou, Xiaoyan Yan.

**Data curation:** Xueqin Yu, Minghua Yuan, Mengxin Yi, Hong Ding, Jun Huang, Wenbing Zou, Xiaoyan Yan.

**Formal analysis:** Xueqin Yu, Minghua Yuan, Mengxin Yi, Hong Ding, Jun Huang, Wenbing Zou, Xiaoyan Yan.

**Funding acquisition:** Xiaoyan Yan.

**Investigation:** Xiaoyan Yan.

**Writing – original draft:** Xiaoyan Yan.

**Writing – review & editing:** Xiaoyan Yan.

## References

[1] Li D Li S Chen Q Xie X. The prevalence of symptomatic knee osteoarthritis in relation to age, sex, area, region, and body mass index in China: a systematic review and meta-analysis. Front Med (Lausanne). 2020;7:304. 32766258 10.3389/fmed.2020.00304 PMC7378378

[2] McAlindon TE Bannuru RR Sullivan MC. OARSI guidelines for the non-surgical management of knee osteoarthritis. Osteoarthritis Cartilage. 2014;22:363–88. 24462672 10.1016/j.joca.2014.01.003

[3] Laufer Y Dar G. Effectiveness of thermal and athermal short-wave diathermy for the management of knee osteoarthritis: a systematic review and meta-analysis. Osteoarthritis Cartilage. 2012;20:957–66. 22659070 10.1016/j.joca.2012.05.005

[4] Berman BM Lao L Langenberg P Lee WL Gilpin AM Hochberg MC. Effectiveness of acupuncture as adjunctive therapy in osteoarthritis of the knee: a randomized, controlled trial. Ann Intern Med. 2004;141:901–10. 15611487 10.7326/0003-4819-141-12-200412210-00006

[5] Wang Z Zhao C Li M. Efficacy and safety of external therapies of traditional Chinese medicine in patients with knee osteoarthritis: a systematic review and network meta-analysis. Rejuvenation Res. 2025;28:248–62. 40511477 10.1089/rej.2025.0039

[6] Kazempour Mofrad R Dadarkhah A Rezasoltani Z Najafi S. Evaluating the efficacy of electroacupuncture compared to physiotherapy in reducing pain and disability in soldiers diagnosed with chondromalacia patella: a randomized clinical trial. Anesth Pain Med. 2024;14:e143688. 40078468 10.5812/aapm-143688 PMC11895788

[7] Núñez-Cortés R Cruz-Montecinos C Vásquez-Rosel A Paredes-Molina O Cuesta-Vargas A. Dry needling combined with physical therapy in patients with chronic postsurgical pain following total knee arthroplasty: a case series. J Orthop Sports Phys Ther. 2017;47:209–16. 28158960 10.2519/jospt.2017.7089

[8] Zhu RX Li X Yan ZB. Efficacy and safety of acupuncture with moxibustion for knee osteoarthritis: a meta-analysis of randomized controlled trials. Syst Rev. 2025;14:15. 39819364 10.1186/s13643-025-02762-x PMC11737046

[9] Wei Y Liu L Ge H. Clinical effect of acupuncture on knee osteoarthritis and its effect on p38 MAPK signaling pathway. Turk J Phys Med Rehabil. 2024;70:335–43. 39679112 10.5606/tftrd.2024.13186 PMC11639502

[10] Huang DE Qin Y Lin MN Lai XL. Clinical efficacy of different waves of electroacupuncture on knee osteoarthritis and its effect on TGF-β1 in joint fluid. Zhongguo Zhen Jiu. 2020;40:370–4. 32275364 10.13703/j.0255-2930.20190422-0005

[11] Wang H Zhang C Gao C. Effects of short-wave therapy in patients with knee osteoarthritis: a systematic review and meta-analysis. Clin Rehabil. 2017;31:660–71. 28118736 10.1177/0269215516683000

[12] Collins NJ Misra D Felson DT Crossley KM Roos EM. Measures of knee function: International Knee Documentation Committee (IKDC) Subjective Knee Evaluation Form, Knee Injury and Osteoarthritis Outcome Score (KOOS), Knee Injury and Osteoarthritis Outcome Score Physical Function Short Form (KOOS-PS), Knee Outcome Survey Activities of Daily Living Scale (KOS-ADL), Lysholm Knee Scoring Scale, Oxford Knee Score (OKS), Western Ontario and McMaster Universities Osteoarthritis Index (WOMAC), Activity Rating Scale (ARS), and Tegner Activity Score (TAS). Arthritis Care Res (Hoboken). 2011;63:S208–28. 22588746 10.1002/acr.20632 PMC4336550

[13] Mansfield CJ Vanetten L Willy R di Stasi S Magnussen R Briggs M. The effects of needling therapies on muscle force production: a systematic review and meta-analysis. J Orthop Sports Phys Ther. 2019;49:154–70. 30501386 10.2519/jospt.2019.8270

[14] Jan MH Chai HM Wang CL Lin YF Tsai LY. Effects of repetitive shortwave diathermy for reducing synovitis in patients with knee osteoarthritis: an ultrasonographic study. Phys Ther. 2006;86:236–44. 16445337

[15] Riddle DL Kong X Jiranek WA. Factors associated with rapid progression to knee arthroplasty: complete analysis of three-year data from the Osteoarthritis Initiative. Joint Bone Spine. 2012;79:298–303. 21727020 10.1016/j.jbspin.2011.05.005

[16] Chen N Wang J Mucelli A Zhang X Wang C. Electro-acupuncture is beneficial for knee osteoarthritis: the evidence from meta-analysis of randomized controlled trials. Am J Chin Med. 2017;45:965–85. 28659033 10.1142/S0192415X17500513

[17] Akyol Y Durmus D Alayli G. Does short-wave diathermy increase the effectiveness of isokinetic exercise on pain, function, knee muscle strength, quality of life, and depression in the patients with knee osteoarthritis? A randomized controlled clinical study. Eur J Phys Rehabil Med. 2010;46:325–36. 20926998

[18] Atamaz FC Durmaz B Baydar M. Comparison of the efficacy of transcutaneous electrical nerve stimulation, interferential currents, and shortwave diathermy in knee osteoarthritis: a double-blind, randomized, controlled, multicenter study. Arch Phys Med Rehabil. 2012;93:748–56. 22459699 10.1016/j.apmr.2011.11.037

[19] Lv ZT Shen LL Zhu B. Effects of intensity of electroacupuncture on chronic pain in patients with knee osteoarthritis: a randomized controlled trial. Arthritis Res Ther. 2019;21:120. 31088511 10.1186/s13075-019-1899-6 PMC6518678

[20] Ho KK Kwok AW Chau WW Xia SM Wang YL Cheng JC. A randomized controlled trial on the effect of focal thermal therapy at acupressure points treating osteoarthritis of the knee. J Orthop Surg Res. 2021;16:282. 33906695 10.1186/s13018-021-02398-2 PMC8077935

[21] Wang Z Wang Y Wang C. Systematic review and network meta-analysis of acupuncture combined with massage in treating knee osteoarthritis. Biomed Res Int. 2022;2022:4048550. 35996547 10.1155/2022/4048550 PMC9392627

[22] Rabini A Piazzini DB Tancredi G. Deep heating therapy via microwave diathermy relieves pain and improves physical function in patients with knee osteoarthritis: a double-blind randomized clinical trial. Eur J Phys Rehabil Med. 2012;48:549–59. 22820824

